# PITX2 Loss-of-Function Mutation Contributes to Congenital Endocardial Cushion Defect and Axenfeld-Rieger Syndrome

**DOI:** 10.1371/journal.pone.0124409

**Published:** 2015-04-20

**Authors:** Cui-Mei Zhao, Lu-Ying Peng, Li Li, Xing-Yuan Liu, Juan Wang, Xian-Ling Zhang, Fang Yuan, Ruo-Gu Li, Xing-Biao Qiu, Yi-Qing Yang

**Affiliations:** 1 Department of Cardiology, Tongji Hospital, Tongji University School of Medicine, Shanghai, China; 2 Division of Medical Genetics, Tongji University School of Medicine, Shanghai, China; 3 Department of Pediatrics, Tongji Hospital, Tongji University School of Medicine, Shanghai, China; 4 Department of Cardiology, Shanghai Tenth People's Hospital, Tongji University School of Medicine, Shanghai, China; 5 Department of Cardiology, Shanghai Chest Hospital, Shanghai Jiao Tong University, Shanghai, China; 6 Department of Cardiovascular Research Laboratory, Shanghai Chest Hospital, Shanghai Jiao Tong University, Shanghai, China; 7 Department of Central Laboratory, Shanghai Chest Hospital, Shanghai Jiao Tong University, Shanghai, China; Medical College of Wisconsin, UNITED STATES

## Abstract

Congenital heart disease (CHD), the most common type of birth defect, is still the leading non-infectious cause of infant morbidity and mortality in humans. Aggregating evidence demonstrates that genetic defects are involved in the pathogenesis of CHD. However, CHD is genetically heterogeneous and the genetic components underpinning CHD in an overwhelming majority of patients remain unclear. In the present study, the coding exons and flanking introns of the *PITX2* gene, which encodes a paired-like homeodomain transcription factor 2essential for cardiovascular morphogenesis as well as maxillary facial development, was sequenced in 196 unrelated patients with CHD and subsequently in the mutation carrier’s family members available. As a result, a novel heterozygous PITX2 mutation, p.Q102X for PITX2a, or p.Q148X for PITX2b, or p.Q155X for PITX2c, was identified in a family with endocardial cushion defect (ECD) and Axenfeld-Rieger syndrome (ARS). Genetic analysis of the pedigree showed that the nonsense mutation co-segregated with ECD and ARS transmitted in an autosomal dominant pattern with complete penetrance. The mutation was absent in 800 control chromosomes from an ethnically matched population. Functional analysis by using a dual-luciferase reporter assay system revealed that the mutant PITX2 had no transcriptional activity and that the mutation eliminated synergistic transcriptional activation between PITX2 and NKX2.5, another transcription factor pivotal for cardiogenesis. To our knowledge, this is the first report on the association of PITX2 loss-of-function mutation with increased susceptibility to ECD and ARS. The findings provide novel insight into the molecular mechanisms underpinning ECD and ARS, suggesting the potential implications for the antenatal prophylaxis and personalized treatment of CHD and ARS.

## Introduction

Congenital heart disease (CHD) is the most prevalent type of birth defect in humans, with an estimated prevalence of 1% among living neonates, and is the most common non-infectious cause of infant morbidity and mortality, accounting for roughly 30% of neonatal demises caused by miscellaneous developmental malformations [1]. Traditionally, various CHDs are categorized as at least 21 distinct entities with specific anatomic lesions, including ventricular septal defect, atrial septal defect, tetraology of Fallot, endocardial cushion defect (ECD), double outlet right ventricular, patent ductus arteriosus, and transposition of the great vessels [1]. Distinct forms of CHDs can occur separately or in combination, leading to reduced exercise performance, degraded quality of life, delayed brain development or brain injury, thromboembolic stroke, pulmonary hypertension, impaired pulmonary function, metabolic disorders, muscle dysfunction, abnormal autonomic nervous activity, infective endocarditis, cardiac enlargement or congestive heart failure, arrhythmias, and sudden cardiac death [2–13]. Obviously, CHD has imposed an enormous economic burden on patients and health care systems, and the socioeconomic burden is anticipated to increase in the future with increasing CHD adults [14,15]. Despite the pronounced clinical importance, the molecular mechanisms underpinning CHD remain poorly understood.

In vertebrates, the heart is the first organ that develops to function. Cardiovascular morphogenesis is a complex, dynamic biological process that requires the orchestration of cardiac cell commitment, differentiation, proliferation and migration, and both environmental and genetic risk factors may interrupt this accurate temporal and spatial cooperation, yielding a wide range of CHD [16–38]. There is increasing evidence that highlights the pivotal role of cardiac transcription factors in embryonic cardiogenesis, and a long list of mutations in the cardiac transcription factor genes, including NK and GATA families, have been implicated in the pathogenesis of CHD [39–65]. However, CHD is of striking genetic heterogeneity and the genetic components predisposing to CHD in an overwhelming majority of patients remain to be identified.

Recently, there is increasing evidence demonstrating that the transcription factor PITX2, a member of the bicoid-like homeodomain family of transcription factors, plays a crucial role in cardiovascular morphogenesis and maxillary facial development. The *PITX2* gene was originally identified as a causative gene for the human Axenfeld-Rieger's syndrome (ARS), which is characterized by eye, teeth, craniofacial and umbilical abnormalities as well as heart defects [66–68]. To date, four different isoforms of PITX2 transcripts, which are generated by differential mRNA splicing and alternative promoter usage, have been identified, of which PITX2a, PITX2b and PITX2c differ only in their amino-termini and exist in human, mouse, chick, zebrafish and xenopus, while the fourth isoform, PITX2d, which lacks most homeodomain along with the entire amino-terminal domain, is detected only in humans. Notably, PITX2c is the predominant transcript in the embryonic and adult heartsof the mouse and human, mainly responsible for cardiogenesis [69–78]. In Xenopus embryos, partial depletion of PITX2c mRNA using chemically modified antisense oligonucleotides resulted in cardiac dysmorphology, including abnormalities of outflow tract, atrial septation and relative atrial-ventricular chamber positioning as well as restriction of ventricular development [79]. In mice, targeted disruption of PITX2c resulted in embryonic lethality with different kinds of congenital cardiovascular malformations, including ECD, atrial isomerism, double-outlet right ventricle, transposition of the great artery and abnormal aortic arch [80,81]. In humans, PITX2cmutations have been causatively associated with isolated congenital heart diseases [82–84]. These findings justified screening PITX2 as a preferred candidate gene for CHD in other cohorts of patients.

## Materials and Methods

### Study participants

In this study, 196 unrelated CHD patients and 400 unrelated individuals with no cardiac structural aberrations were enrolled from the Chinese Han population. The available relatives of an index patient with an identified PITX2 mutation were also included. All participants underwent detailed clinical evaluation, which included individual and familial histories, comprehensive physical examination, and echocardiography with color flow Doppler. The patients also underwent chest X-ray, electrocardiogram or cardiac catheterization examination when there was a strong clinical indication. Medical records of the deceased or unavailable relatives of a mutation carrier were also reviewed. The patients with known chromosomal abnormalities were excluded from the study. Peripheral venous blood samples were taken from all participants. This study conformed to the ethical guidelines of the Declaration of Helsinki. The study protocol was reviewed and approved by the ethics committee of Tongji Hospital, Tongji University (the ethical approval number for cases and controls: LL(H)-09-07; the date for the approval: July 27, 2009). Written informed consent was signed by participants or their guardians prior to study.

### Genetic analysis of human *PITX2*

Genomic DNA was isolated from peripheral blood leukocytes using the Wizard Genomic DNA Purification Kit (Promega, Madison, WI, USA). The coding regions and splice junction sites of the *PITX2* gene was sequenced initially in 196 unrelated patients with CHD, and genotyping *PITX2* was performed subsequently in the available relatives of a mutation carrier and 400 unrelated control individuals. The referential genomic DNA sequence of *PITX2* was derived from GenBank (accession no. NC_000004), which was at the National Center for Biotechnology Information (NCBI; http://www.ncbi.nlm.nih.gov/). The primer pairs used to amplify the coding exons and intron–exon boundaries of *PITX2* by polymerase chain reaction (PCR) were shown in Table 1. The PCR was performed and the PCR product was sequenced as previously described [82]. A sequence variation was verified by re-sequencing an independent PCR-amplified product from the same subject. Additionally, for an identified sequence variant, the Exome Variant Server (EVS; http://evs.gs.washington.edu/EVS) and NCBI’s single nucleotide polymorphism (SNP; http://www.ncbi.nlm.nih.gov/SNP) databases were queried to confirm its novelty.

**Table 1:** The primers to amplify the coding exons and flanking introns of *PITX2*.

Exon	Forward primer (5′ to 3′)	Reverse primer (5′ to 3′)	Amplicon (bp)
2	GAGGCTAGGCTGGAGATGCT	CCACTGGCGATTTGGTTCTG	385
3	TTGCTCTTTGTCCCTCTTTC	CCAGAGGCGGAGTGTCTAAG	399
4	CAGCTTGGCTTGAGAACTCG	TGACTTCCTTGGGGCGAGAG	442
5	CAGCTCTTCCACGGCTTCTG	GCTGCCTTCCACATTCTCTC	387
6	AATCTGCACTGTGGCATCTG	AGTCTTTCAAGGGCGGAGTT	677

### Alignment of multiple PITX2 protein sequences across species

Multiple amino acid sequences of the PITX2 proteins from various species were aligned using the online MUSCLE program, version 3.6 (http://www.ncbi.nlm.nih.gov/).

### Plasmids and site-directed mutagenesis

The expression plasmid PITX2c-pcDNA4 was a kind gift from Georges Christé at Physiopathologie des Troubles du RythmeCardiaque, Faculté de Pharmacie de Lyon, Université Lyon 1, France. The recombinant expression plasmid NKX2.5-pEFSA and the atrial natriuretic factor (ANF)-luciferase reporter plasmid (ANF-luc), which contains the 2600-bp 5’-flanking region of the *ANF* gene and expresses Firefly luciferase, were kindly provided by Dr. Ichiro Shiojima, from the Department of Cardiovascular Science and Medicine, Chiba University Graduate School of Medicine, Chuo-ku, Chiba, Japan. The procollagen lysyl hydroxylase (PLOD1) promoter plasmid PLOD1-luc, which contains the nucleotides from -60 to -3180 of the *PLOD1* gene, was constructed as described previously [85]. The PITX2a and PITX2b isoforms were PCR-amplified from cDNA clones as described previously [86] and inserted into the pcDNA4 plasmid (Invitrogen, Carlsbad, CA, USA), respectively. The identified mutation Q102X, or Q148X, or Q155X was introduced into the wild-type PITX2a, or PITX2b, or PITX2c, respectively, by using a QuickChange II XL Site-Directed Mutagenesis Kit (Stratagene, La Jolla, CA, USA) with a complementary pair of primers. Each of themutants was sequenced to confirm the desired mutation and to exclude any other sequence variations.

### Luciferase reporter gene assays

Chinese hamster ovary (CHO) cells were seeded in 12-well plates and cultured in Dulbecco’s Modified Eagle Medium supplemented with 10% fetal bovine serum, 100 mg/ml penicillin, and 100 mg/ml streptomycin in a humidified atmosphere containing 5% CO_2_ at 37°C. Cell transfections were performed 24 h after plating, with Lipofectamine 2000 Transfection Reagent (Invitrogen) according to the manufacturer’s protocol. The ANF-luc construct and an internal control reporter plasmid pGL4.75 (hRluc/CMV, Promega), which expresses Renilla luciferase, were used in transient transfection assays. CHO cells were transfected with 2 μg of wild-type PITX2–pcDNA4 or mutant PITX2–pcDNA4 or empty vector pcDNA4, 2.0 μg of ANF-luc reporter construct, and 0.04 μg of pGL4.75 control reporter vector. For co-transfection experiments, 1 μg of wild-type PITX2–pcDNA4, 1 μg of mutant PITX2–pcDNA4, 2.0 μg of ANF-luc, and 0.04 μg of pGL4.75 were used. Transfected cells were harvested 24 h after transfection, then lysed and assayed for reporter activities. Firefly luciferase and Renilla luciferase activities were measured with the Dual-Glo luciferase assay system (Promega). The activity of the ANF promoter was presented as fold activation of Firefly luciferase relative to Renilla luciferase. Three independent experiments were conducted in triplicate for wild-type and mutant PITX2a, or PITX2b, or PITX2c, and results are representative of three separate experiments.

For the analysis of the synergistic transcriptional activation between PITX2 and NKX2.5 [87], another transcription factor crucial for normal cardiovascular development [40–48], CHO cells were grown and transfected with 2μg of wild-type or mutant PITX2–pcDNA4, alone or together with 2μg of wild-type NKX2.5-pEFSA, 5μg of PLOD1-luc, and 0.04 μg of pGL4.75 using Lipofectamine 2000 Transfection Reagent (Invitrogen).

### Statistical analysis

The significance of differences in luciferase activity was analyzed using the unpaired Student’s *t* test. A two-tailed *P* value less than 0.05 was considered to be statistically significant.

## Results

### Baseline characteristics of the study subjects

A cohort of 196 unrelated patients with CHD was clinically investigated in contrast to a total of 400 ethnically-matched unrelated controls. All the participants had no established environmental risk factors for CHD, such as maternal illness and drug use in the first trimester of pregnancy, parental smoking and long-term exposure to toxicants as well as ionizing radiation. The control individuals had no evidence of organic cardiac diseases, and their echocardiographic results were normal. The baseline clinical characteristics of the 196 CHD patients are summarized in Table 2.

**Table 2:** Baseline clinical characteristics of the 196 unrelated patients with congenital heart disease.

Variable	Statistic
Male gender (%)	102 (52.0)
Age (years)	5.2 ± 2.4
Positive family history (%)	36 (18.4)
Prevalence of different types of CHD	
Isolated CHD (%)	105 (53.6)
VSD (%)	32 (16.3)
ASD (%)	27 (13.8)
PDA (%)	20 (10.2)
ECD (%)	6 (3.1)
AS (%)	5 (2.6)
PA (%)	5 (2.6)
CoA (%)	4 (2.0)
PS (%)	3 (1.5)
TA	2 (1.0)
HLHS (%)	1 (0.5)
Complex CHD (%)	72 (36.7)
TOF (%)	28 (14.3)
DORV + VSD (%)	17 (8.7)
ECD + TGA (%)	14 (7.1)
TA + VSD (%)	9 (4.6)
TGA +VSD (%)	4 (2.0)
Others (%)	19 (9.7)
Incidence of arrhythmia	
Atrial fibrillation (%)	16 (8.2)
Atrioventricular block (%)	8 (4.1)
Treatment	
Surgical repair (%)	118 (60.2)
Catheter-based closure (%)	57 (29.1)
Follow-up (%)	21 (10.7)

CHD, congenital heart disease; VSD, ventricular septal defect; ASD, atrial septal defect; PDA, patent ductus arteriosus; ECD, endocardial cushion defect; AS, aortic stenosis; PA, pulmonary atresia; CoA, coarctation of the aorta; PS, pulmonary stenosis; TA, truncusarteriosus; HLHS, hypoplastic left heart syndrome; TOF, tetralogy of Fallot; DORV, double outlet of right ventricle; TGA, transposition of great arteries.

### Identification of a novel PITX2 mutation

**Fig 1:**
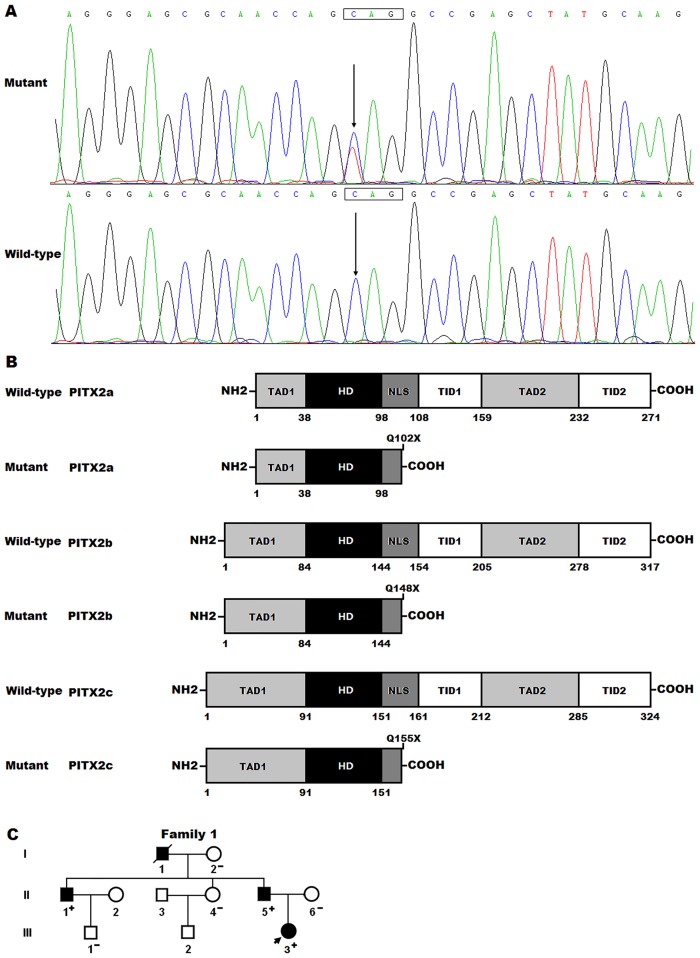
PITX2 mutation associated with endocardial cushion defect and Axenfeld-Rieger syndrome. (A) Sequence electropherograms showing the heterozygous *PITX2* mutation compared with its control. The arrow indicates the heterozygous nucleotides of C/T in the proband (mutant) or the homozygous nucleotides of C/C in the corresponding control individual (wild-type). The rectangle signifies the nucleotides comprising a codon of PITX2. (B) Schematic diagrams showing the structural domains of wild-type and mutant PITX2 proteins with the disease related mutation indicated. The mutation found in patients with endocardial cushion defect and Axenfeld-Rieger syndrome is shown above the structural domains of the mutant PITX2 proteins. NH2 denotes amino-terminus; TAD1, transcriptional activation domain 1; HD, homeodomain; NLS, nuclear localization signal; TID1, transcriptional inhibitory domain 1; TAD2, transcriptional activation domain 2; TID2, transcriptional inhibitory domain 2; COOH, carboxyl-terminus. (C) Pedigree structure of the family with endocardial cushion defect and Axenfeld-Rieger syndrome. Family members are identified by generations and numbers. Square indicates male family member; circle, female member; symbol with a slash, the deceased member; closed symbol, affected member; open symbol, unaffected member; arrow, proband; “+”, carrier of the heterozygous mutation; “–”, non-carrier.

By sequencing of PITX2 in the 196 patients, a heterozygous sequence variation was identified in one patient, with a mutational prevalence of about 0.51%. Specifically, a substitution of thymine for cytosine at the first nucleotide of codon 102 of PITX2a (c.304C>T), or codon 148 of PITX2b (c.442C>T), or codon 155 of PITX2c (c.463C>T), predicting the transition of glutamine-encoding codon to a stop codon at amino acid 102 for PITX2a (p.Q102X), or 148 for PITX2b (p.Q148X), or 155 for PITX2c (p.Q155X), was identified in an ECD patient with positive family history. The sequence electropherograms showing the identified nonsense PITX2 variation compared with the corresponding control sequence are shown in Fig 1A. The schematic diagrams showing the structural domains of the wild-type and mutant PITX2 proteins are presented in Fig 1B. The variation was neither observed in 800 control chromosomes nor reported in the EVS’s and NCBI’s SNP databases, which were consulted again on September 1, 2014. Genetic screening of the mutation carrier’s family members demonstrated that the variation was present in all affected family members available, but absent in unaffected family members examined. Analysis of the pedigree showed that in the family the mutation co-segregated with ECD transmitted as an autosomal dominant trait with complete penetrance. The pedigree structure of the family is illustrated in Fig 1C. Besides, the proband (III-3) had also transposition of the great arteries, and her father (II-5) and uncle (II-1) had also mitral valve cleft and right aortic arch. Interestingly, all the mutation carriers had also oligodontia, maxillary hypoplasia and iris hypoplasia, and the proband (III-3) and her father (II-5) had also congenital umbilical hernia, a phenotype of Axenfeld-Rieger syndrome (ARS). The phenotypic characteristics and results of genetic screening of the affected pedigree members are listed in Table 3.

**Table 3:** Phenotypic characteristics and status of PITX2 mutation of the affected pedigree members.

Subject information			Phenotype		Genotype
Identity	Gender	Age (years)	Cardiac defects	Extracardiac defects	PITX2 mutation
I-1	M	50^a^	ECD	OD, MH, IH	NA
II-1	M	31	ECD, RAA, MVC	OD, MH, IH	+/–
II-5	M	26	ECD, RAA, MVC	OD, MH, IH, UH	+/–
III-3	F	1	ECD, TGA	OD, MH, IH, UH	+/–

M, male; F, female; ECD, endocardial cushion defect; MVC, mitral valve cleft; RAA, right aortic arch; TGA, transposition of the great arteries; OD, oligodontia; MH, maxillary hypoplasia; IH, iris hypoplasia; UH, umbilical hernia; NA, not available; +/–, heterozygote.

^a^Age at death.

### Multiple alignments of PITX2 protein sequences across species

**Fig 2:**
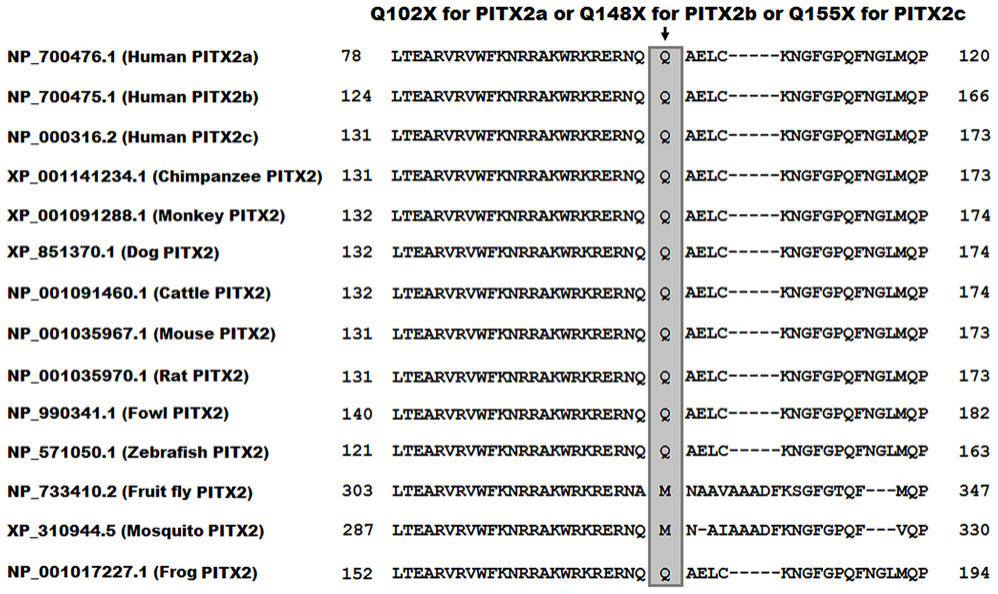
Alignment of multiple PITX2 amino acid sequences among species. The altered amino acid of p.Q102 for PITX2a, or p.Q148 for PITX2b, or p.Q155 for PITX2c is completely conserved evolutionarily among vertebrates.

A cross-species alignment of PITX2 protein sequences displayed that the altered amino acid, p.Q102 for PITX2a, or p.Q148 for PITX2b, or p.Q155 for PITX2c, was completely conserved evolutionarily among all vertebrates (Fig 2).

### Transactivational activity of the mutant PITX2

**Fig 3:**
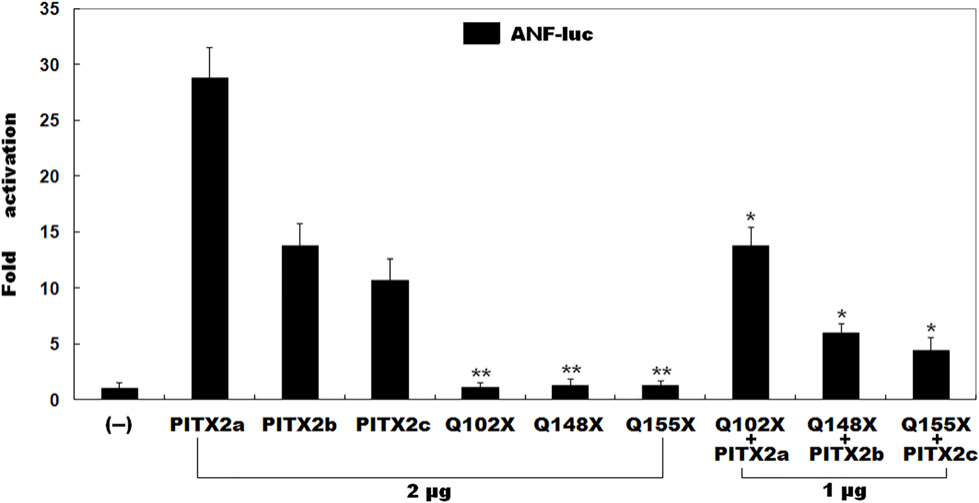
Transactivational defects caused by PITX2 mutation. Transcriptional activation of atrial natriuretic factor promoter driven luciferase reporter in CHO cells by wild-type or mutant PITX2, alone or in combination, showed that the mutant PITX2 did not transactivate gene expression. Data are derived from three independent experiments repeated in triplicate. Mean fold activation and standard deviations are shown. ** and * represent *P*<0.001 and *P*<0.01, respectively, when compared with wild-type PITX2.

As shown in Fig 3, the wild-type PITX2a, PITX2b and PITX2c activated the *ANF* promoter by ~29-fold, ~14-fold and ~11-fold, respectively; whereas the same amount (2 μg) of mutant PITX2a, PITX2b or PITX2c activated the *ANF* promoter by ~1-fold. When the same amount of wild-type *PITX2* (1 μg) was cotransfected with mutant *PITX2* (1 μg), the induced activation of the *ANF* promoter was ~14-fold for PITX2a, ~6-fold for PITX2b and ~4-fold for PITX2c. These results suggest that the mutant PITX2 has no transactivational activity when compared with its wild-type counterpart.

### Synergistic transcriptional activity between mutant PITX2 and NKX2.5

**Fig 4:**
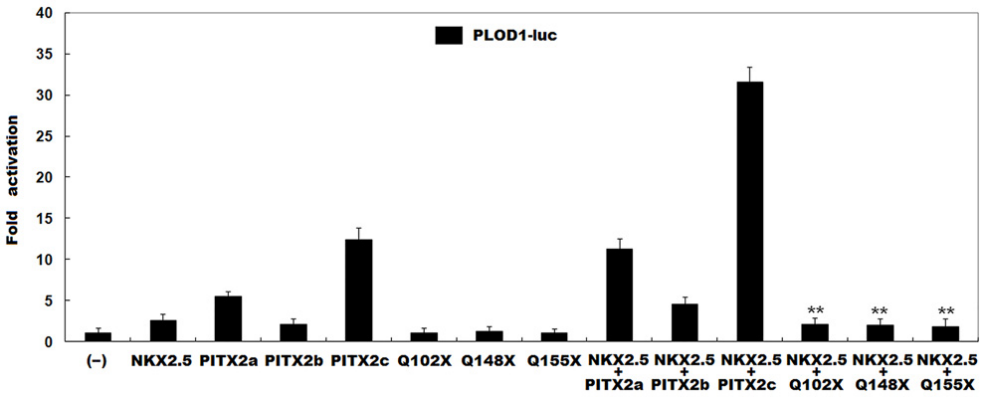
No synergistic transcriptional activation between NKX2.5 and mutant PITX2. The synergistic transactivation of the *PLOD1* promoter in CHO cells by NKX2.5 and mutant PITX2 was eliminated by the mutation. All data are derived from three independent experiments repeated in triplicate. Mean fold activation and standard deviations are shown. ** represents *P*<0.001, when compared with NKX2.5 plus wild-type PITX2.

As shown in Fig 4, in the presence of 2μg of wild-type *NKX2*.*5*, 2μg of wild-type PITX2a, PITX2b and PITX2c activated the *PLOD1* promoter by ~11-fold, ~5-fold and ~32-fold, respectively; while the same amount (2μg) of Q102X-mutant PITX2a, or Q148-mutant PITX2b or Q155X-mutant PITX2c activated the *PLOD1* promoter by ~2-fold, indicating that the mutation blocks the synergistic transactivational activity between PITX2 and NKX2.5.

## Discussion

In the current study, a novel heterozygous mutation in the *PITX2* gene, p.Q102X for PITX2a, p.Q148X for PITX2b, or p.Q155X for PITX2c, was identified in a family with congenital ECD and ARS. Genetic analysis of the pedigree showed that the nonsense mutation was transmitted in an autosomal dominant pattern with complete penetrance. The mutation, which was absent in the 800 reference chromosomes, altered the amino acid highly conserved evolutionarily among vertebrates. Functional assays unveiled that each isoform of the mutant PITX2 lost the ability to transactivate the *ANF* and *PLOD1* promoters and that the mutation eliminated the synergistic transcriptional activation between PITX2 and NKX2.5. Hence, it is very likely that genetically defective PITX2 confers enhanced susceptibility to ECD and ARS in these mutation carriers.

It has been revealed that PITX2 is abundantly expressed in the developing hearts, craniofacial organs, and abdominal wall, especially in myocardium related to endocardial cushions of the atrioventricular canal, and functions to mediate multiple target genes that are amply expressed during embryogenesis, including *ANF* and PLOD1 [66–74]. Therefore, the transcriptional effect of a mutant PITX2 may be characterized by using the *ANF* and PLOD1 promoters. In this study, functional analyses demonstrated that the mutation identified in patients with ECD and ARS abolished the transcriptional activation of ANF- or PLOD1-driven luciferase reporter by PITX2 and eliminated the transcriptionally synergistic activation between PITX2 and NKX2.5, indicating that functionally impaired PITX2 is potentially an alternative molecular mechanism underpinning CHD and ARS.

Previous studies have established that multiple important genes are transcriptionally regulated by PITX2c during cardiovascular development [87], and mutations in several target genes, such as NKX2.5 and GATA4, have been causally implicated in CHD including ECD [40–48,51–58]. Therefore, mutated PITX2c may increase the vulnerability to CHD by altering the expressions of such cardiac-specific target genes.

In humans, PITX2c mutations have been implicated in the pathogenesis of other CHDs. Wang and co-workers [82] screened PITX2c in 382 unrelated patients with CHDs and found two heterozygous mutations, p.W147X and p.N153D, in two patients with CHD, respectively, including a one-year-old male patient with double outlet right ventricle in combination with ventricular septal defect and a four-year-old female patient with isolated ventricular septal defect. Yuan et al. [83] scanned PITX2c in 150 unrelated patients with CHDs and identified two novel heterozygous PITX2c mutations, p.H98Q and p.M119T, in two patients with atrial septal defects, respectively. Wei and colleagues [84] also sequenced PITX2c in 170 unrelated neonates with CHDs and detected two novel heterozygous PITX2c mutations, p.R91Q and p.T129S, in two unrelated newborns with transposition of the great arteries and ventricular septal defect, respectively. Functional analysis demonstrated that all the above-mentioned PITX2c mutations were consistently associated with significantly diminished transcriptional activity [82–84]. In this study, a novel PITX2 loss-of-function mutation is identified in patients with ECD and ARS, thus expanding the phenotypic spectrum linked to PITX2 mutation.

Association of genetically compromised PITX2 with enhanced susceptibility to ECD has been demonstrated in animal models [79–81]. In mice, PITX2 deficiency results in complicated cardiac defects, including atrial septal defect, ventricular septal defect, ECD, hypoplasia of the right ventricle, and failure to form normal cardiac valves [81]. Further studies shows that ablation of PITX2 results in distortion, rather than loss, of muscle anlagen, suggesting that its function becomes critical during the colonization of, and/or fiber assembly in, the anlagen. In addition, myogenic cells lacking PITX2 are smaller and more symmetrical with decreased motility, which may prevent proper assembly of higher-order fibers within anlagen [88]. Nevertheless, PITX2c expression in mesenchymal cushion cells remains a controversial topic. Furtado and colleagues [70] reported that in mice PITX2c was expressed in trabecular and septal, as well as non-trabecular, myocardium, and had a strong expression bias in myocardium associated with individual endocardial cushions of the atrioventricular canal and outflow tract, which are essential for cardiac septation. Two other groups [80,89] also reported the expression of PITX2c in these structures. Fate-mapping studies using a *PITX2 cre recombinase* knock-in allele showed that daughters of *PITX2*-expressing cells populated the right and left ventricles, atrioventricular cushions and valves and pulmonary veins. In *PITX2* mutant embryos, descendents of *PITX2*-expressing cells failed to contribute to the atrioventricular cushions and valves and the pulmonary vein, resulting in abnormal morphogenesis of these structures [80]. However, lineage-tracing studies in mice showed that myocardium did not transform into mesenchyme in cushions [90]. In humans, PITX2c was expressed predominantly in left atria, with lower levels in right atrium and left and right ventricles [72]. Due to pronounced spatial and temporal difference in gene expression even for the same species, further work will be necessary to clarify this issue, especially for all isoforms of PITX2 in human heart.

Up to now, in humans mutated PITX2 has been linked to type 1 ARS [66–68], type 2 iridogoniodysgenesis [91], Peters’ anomaly [92], ring dermoid of cornea [93], various congenital heart diseases [16,82–84], and atrial fibrillation [94–97]. In this study, a novel PITX2 mutation was linked to atypical ARS with ECD being the main phenotype. The remarkable phenotypic diversities caused by PITX2 mutations may be explained as follows. Firstly, different genetic backgrounds, including possibly common SNPs altering disease susceptibility, contribute to the variable phenotypes. Secondly, distinct epigenetic modifiers may account for the significant phenotypic heterogeneity among these mutation carriers. Thirdly, delayed penetrance or incomplete penetrance may also be responsible for the discrepant clinical expressivity. Finally, mutations as found in this study may be merely a genetic risk factor predisposing to a disease, rather than a direct cause, and environmental risk factors may be required for the onset of the disease [98].

## Conclusions

In conclusion, this study firstly links PITX2 loss-of-function mutation to ECD and ARS, which provides novel insight into the molecular mechanisms of CHD and ARS, implying potential implications in antenatal prophylaxis and personalized treatment of CHD and ARS.
